# Genomic Characterization and Functional Evaluation of *Eurotium cristatum* EC-520: Impacts on Colon Barrier Integrity, Gut Microbiota, and Metabolite Profile in Rats

**DOI:** 10.3390/foods14091569

**Published:** 2025-04-29

**Authors:** Huini Wu, Xiuping Wang, Xiangrui Kong, Ruiyang Shan, Song Peng, Mengshi Zhao, Changsong Chen, Wenquan Yu, Zhaolong Li

**Affiliations:** 1 Institute of Animal Husbandry and Veterinary Medicine, Fujian Academy of Agricultural Sciences, Fuzhou 350013, China; 5220330098@fafu.edu.cn (H.W.); pengsong@faas.cn (S.P.); 13375001253@163.com (M.Z.); 2 Tea Research Institute, Fujian Academy of Agricultural Sciences, Fuzhou 350013, China; wangxiuping@faas.cn (X.W.); kongxiangrui_2008@163.com (X.K.); fjnkycys@163.com (R.S.); ccs6536597@163.com (C.C.)

**Keywords:** *Eurotium cristatum*, EC-520, colon, microbiota, metabolomics

## Abstract

*Eurotium cristatum* (EC), the dominant fungus in Fuzhuan brick tea, has significant applications in food fermentation and pharmaceutical industries, exhibiting probiotic properties, but further investigation of its intestinal benefits is required. This study characterized the EC-520 strain through whole genome sequencing and evaluated its effects on rat colons using histomorphology, 16S rRNA sequencing, and untargeted metabolomics. The genomic analysis revealed that EC-520 possessed a 28.37 Mb genome distantly related to *Aspergillus flavus*. The 16S results demonstrated that EC-520 significantly increased the abundance of *Bacteroidota* (*p* < 0.05) while decreasing the *Proteobacteria* and *Firmicutes/Bacteroidota* ratio (the F/B ratio); at the genus level, it elevated *Muribaculaceae* and *Clostridia_UCG-014* while reducing harmful bacteria. The metabolomic results showed that EC-520 also significantly altered tryptamine, caproic acid, isocaproic acid, and erucic acid (*p* < 0.05). Additionally, the Spearman’s correlation analysis revealed that *Muribaculaceae_unclassified* and *Clostridia_UCG-014_unclassified* were significantly positively correlated with tryptamine, caproic acid, isocaproic acid, and erucic acid. Therefore, this study suggested that EC-520 enhanced the colon barrier and increased the abundance of *Muribaculaceae_unclassified* and *Clostridia_UCG-014_unclassified*, thus promoting the secretion of tryptamine and affecting the release of 5-hydroxytryptamine (5-HT). It also promoted the secretion of certain fatty acids, enhancing the balance of the colonic microbiota. This study provides a new view for a comprehensive understanding of EC’s regulatory role in the colon.

## 1. Introduction

*Eurotium cristatum* (EC), a holomorphic ascomycete fungus and the predominant microbial constituent (“Golden Flower Fungus”) in Fuzhuan brick tea (FBT), represents a taxonomically distinct species, formally classified in 1990 as *Eurotium cristatum* (anamorph: *Aspergillus spiculosus*; synonym: *Aspergillus cristatus*) [1,2].

During the fermentation of FBT, EC biosynthesizes diverse bioactive metabolites that confer distinctive organoleptic properties (notably a mellow taste and a fungal, floral aroma) while demonstrating multifunctional activities [3]. These include (i) enhancements in antioxidants [4,5,6,7] and (ii) enzymatic activity [8]; (iii) gut microbiota modulation via a reduction in the F/B ratio and enrichment in probiotic taxa (e.g., *Faecalibacterium*), coupled with antimicrobial metabolite production [9,10]; and (iv) therapeutic potential, as evidenced by hypolipidemic compounds (theabrownins, lovastatin, novel alkaloids) [11,12], antitumor activity against various carcinomas [13], and anti-inflammatory benzaldehyde derivatives [14]. These multifaceted properties position EC as a scientifically significant microbe, with its applications spanning from functional foods to biomedical interventions.

The intestinal microbial community serves as a vital regulator of organismal health, modulating metabolic functions, immune regulation, and barrier function [15]. As a beneficial fungus from FBT, EC has attracted increasing attention in recent years due to its multifaceted regulatory effects on gut health. Studies demonstrated that EC markedly altered gut microbial profiles, enhancing populations of beneficial taxa (*Lactobacillus* and *Ruminococcus_NK4A214_group*) while suppressing pathogenic genera (*Bacteroidota* and *Turicibacter*), thus promoting microbial homeostasis. Regarding metabolic modulation, EC enhanced short-chain fatty acid (SCFA) biosynthesis—particularly acetate, propionate, and butyrate—via the bioactive compounds derived from its fermentation. Concurrently, it ameliorated dyslipidemia in obese animal models, markedly lowering serum total cholesterol (TC), triglyceride (TG), and low-density lipoprotein cholesterol (LDL-C) levels. Furthermore, EC enhanced intestinal barrier function, as its exopolysaccharides helped maintain the barrier’s integrity by regulating epithelial cell metabolism and immune responses, while also exhibiting anti-inflammatory and immunomodulatory effects [16,17]. Although existing studies have confirmed EC’s anti-obesity and anti-inflammatory properties, the biological functions of different strains are not exactly the same (its biological function is closely related to its gene characteristics [18]), and its precise mechanisms of action remain incompletely understood.

To reveal its detailed mechanisms better, the present study isolated the strain EC-520 from FBT and explored the genomic information and species phylogenetic relationships for EC-520 through purification and a whole genome sequencing analysis, which laid the foundation for exploring its functional genes. Then, the effects of EC-520 on the histological morphology of the colon, the colon barrier, and the microbiota and metabolites of rats were investigated, demonstrating the role of EC-520 in the colon microecosystem and providing a scientific basis for its further application.

## 2. Materials and Methods

### 2.1. The Strain, Materials, and Instruments

EC-520 was isolated from Fuzhuan brick tea and stored by the Guangdong Microbiological Strain Preservation Center. The strain preservation number was GDMCC 64205.

Potato dextrose agar (PDA) medium and potato dextrose broth (PDB) medium were purchased from Qindao Hope Bio-Technology Co., Ltd. (Qindao, China). The DNA extraction kit was purchased from Tiangen Biochemical Technology Co., Ltd. (Beijing, China). The RNA extraction kit and PrimeScript Reverse Transcription Master Mix were purchased from TaKaRa Bio. Inc., Shiga, Japan. The 4% polyformaldehyde, hematoxylin, eosin, Alcian blue, periodic acid, peroxidase, phosphate-buffered saline (PBS), goat serum, anti-Mucin-2 (Muc2), anti-Zonula occludens-1 (ZO-1), antibody–horseradish peroxidase (HRP) polymer, 3,3′-Diaminobenzidine tetrahydrochloride, ethyl ether, ethanol, Ethylenediaminetetraacetic acid (EDTA, pH 8.0), 10% donkey serum, goat anti-rabbit immunoglobulin G (IgG), anti-Interleukin-1β (IL-1β) (1:200), anti-Interleukin-6 (IL-6) (1:800), anti-Interleukin-10 (IL-10) (1:200), anti-Tumor necrosis factor-α (TNF-α) (1:500), and 4′,6-diamidino-2-phenylindole (DAPI) were all purchased from Servicebio Technology Co., Ltd. (Wuhan, China). HPLC-grade acetonitrile, ammonia hydroxide, ammonium acetate, and methanol were purchased from Fisher Scientific (Fair Lawn, NJ, USA). Agarose was purchased from Sigma Chemical Co., Ltd. (St. Louis, MO, USA).

This research employed a variety of tools, such as the BioSpec-nano UV-Vis spectrophotometer (Shimadzu, Kyoto, Japan), electrophoresis equipment (Shanghai Sinopharm Chemical Reagent Co., Ltd., Shanghai, China), a C-1000 PCR amplifier, and the CFX Connect fluorescent quantitative PCR device (Bio-Rad Laboratories, Inc., Hercules, CA, USA), along with the Tanon 2500 gel imaging system (Tanon Science & Technology Co., Ltd., Shanghai, China). In addition to these, this study incorporated the SS-325 high-pressure sterilizer (Tomy Digital Biology Co., Ltd., Tokyo, Japan), an ultra-low-temperature freezer (Thermo Fisher Scientific, Waltham, MA, USA), a 5418 low-speed centrifuge, a range of pipettes (Eppendorf AG, Hamburg, Germany), the Milli-QA10 ultrapure water system (Merck Millipore, Burlington, MA, USA), and the F80C ice maker (ICEMATIC S.p.A., Bregnano, CO, Italy). Moreover, this investigation featured a THZ-98 constant-temperature shaker (Shanghai Yiheng Instrument Co., Ltd., Shanghai, China), an electric incubator with temperature control, a clean bench, a standard refrigerator, and additional small household instruments.

### 2.2. The Genomic Analysis

#### 2.2.1. The Isolation and Purification of EC-520

Under sterile conditions, the asci of EC from FBT were directly picked out and inoculated into PDA medium to culture at 28 °C. When the colony became yellow, we picked out an appropriate amount of bacteria using a sterile inoculating loop for plate streaking. This was streaked multiple times until the strain was initially purified. We extracted a single golden colony from the plate and inoculated it into the PDB medium. This was incubated in a shaker at 28 °C for 20 h. Then, we collected the mycelium and extracted the DNA using a fungal DNA extraction kit. The extraction steps were carried out according to the kit’s instructions. The purity and concentration of DNA were measured using the BioSpec-nano ultraviolet–visible spectrophotometer and agarose gel electrophoresis.

For the preparation of the EC-520 spore suspension, we inoculated EC-520 into the prepared PDA medium. After culturing it at 28 °C for 4 d, we scraped off the mycelium and transferred it into a triangular flask containing 100 mL of sterile water. This was shaken at 28 °C and 120 r/min for 30 min. We counted the spores using a hemocytometer and diluted the solution to three gradients of spore concentrations (referred to in previous studies [19]): 1 × 10^8^ CFU/mL (the high dose), 1 × 10^6^ CFU/mL (the medium dose), and 1 × 10^4^ CFU/mL (the low dose). These were stored at 4 °C for subsequent studies.

#### 2.2.2. Sequencing, Assembly, and Annotation of the EC-520 Genome

The qualified genomic DNA of EC-520 was sent to Annoroad Gene Technology Co., Ltd. (Beijing, China) for PacBio Sequel II sequencing. A high-quality region finder (HQRF) and the SNR (Signal–Noise Ratio) were used to filter the data. SMRTlink was used to process subreads. Hifiasm was used to assemble reads. BUSCO was used to assess the assembly. RepeatMasker v1.323, RepeatProteinMask v1.36, and RepeatModeler open-1.0.8 were used to predict repeat sequences. Gene structure predictions were performed using PASA v2.1, Augustus v3.3, and SNAP 38926. The genes were annotated through the NCBI and UniProt. We used tRNAscan-SE to predict the tRNA in the genome sequences. Visualization of the genome assembly was performed using Circos. A phylogenetic analysis was carried out between EC-520 and 23 *Aspergillus* species (downloaded from the NCBI) using tools such as mash, ape, and iTOL.

### 2.3. Animal Experiments

#### 2.3.1. The Animals and Treatments

Forty healthy male Sprague-Dawley rats weighing 100 ± 5 g were purchased from Wu’s Laboratory Animal Co., Ltd. (Fuzhou, China). This study was approved by the Institutional Animal Care and Use Committee at Fujian Academy Agricultural and Sciences (202307FJ010). The rats were housed under a 12 h/12 h light–dark cycle at 20–24 °C and 55–65% humidity. After one week of adaptation, they were randomly divided into 4 groups (n = 10/group): the normal control (CK), high-dose EC-520 (T1), medium-dose EC-520 (T2), and low-dose EC-520 (T3). The CK group received distilled water, while T1-T3 received the EC-520 spore suspensions (2.0 mL/200 g body weight) daily. Their body weight was recorded weekly, while they had free access to food and water.

#### 2.3.2. Sample Collection

After 28 d of feeding and 16 h of fasting, the rats were euthanized through cervical dislocation. Their brains, hearts, livers, spleens, kidneys, and lungs were assessed for lesions in the pathological autopsy. The organ index was calculated as follows: organ weight (g)/body weight (g) × 100. Their mid-colon tissue (1 cm) was fixed in 4% PFA; the remaining colon samples were stored at −80 °C. Their feces (1–2 g) was collected, frozen in liquid nitrogen, and stored at −80 °C.

### 2.4. The Comprehensive Assessment of Colonic Barrier Integrity

#### 2.4.1. Histopathological and Morphological Observations

Hematoxylin and eosin staining (H&E): The colon tissue was fixed in 4% PFA, paraffin-embedded, sliced, xylene-soaked, and rehydrated. It was then stained with hematoxylin (1–3 min), differentiated, blued, eosin-stained (1–2 min), dehydrated, cleared, and mounted. It was observed under a microscope.

Alcian blue–periodic acid–Schiff staining (AB-PAS): The colon tissue was fixed, paraffin-embedded, sliced, and rehydrated. It was stained with Alcian blue (30 min) and oxidized with periodic acid (5 min); Schiff’s reagent was applied (10–20 min); and it was restained with hematoxylin (1 min), dehydrated, cleared, and mounted. Goblet cells/positive areas were quantified using ImageJ.

The immunohistochemical (IHC) analysis: The distal colon tissue was fixed, paraffin- embedded, sliced, dewaxed, and rehydrated. Antigen retrieval was performed, and it was blocked using peroxidase and washed in PBS. Goat serum was applied, and the tissue was incubated (37 °C) with Anti-Muc2/Anti-ZO-1 (1:500, 4 °C overnight) and HRP–secondary antibody (37 °C), developed using DAB, restained with hematoxylin, dehydrated, cleared, and mounted. Muc2/ZO-1 was quantified in 5 fields/section (20×) using ImageJ 1.54i (NIH, Bethesda, MD, USA & LOCI, Madison, WI, USA).

#### 2.4.2. Cytokine Immunofluorescence Analysis of the Colon Tissue

The colon tissue was paraffin-embedded, sliced, degreased, and gradient-dehydrated. Sections were microwave-heated with EDTA (pH 8.0) for antigen retrieval, cooled, and washed with PBS. They were then treated with 10% donkey serum (30 min, RT), washed with PBS, and incubated overnight at 4 °C with the primary antibodies IL-1β (1:200), IL-6 (1:800), IL-10 (1:200), and TNF-α (1:500). After washing them with PBS, the sections were incubated with goat anti-rabbit IgG (50 min, RT, dark), washed again with PBS, DAPI-stained, and imaged using a fluorescence microscope. ImageJ was used to quantify the staining intensity.

#### 2.4.3. Quantitative Analysis of the Protein Expression in the Colon Barrier

Total RNA was extracted from the colon tissue using an RNA kit, with the concentration/purity measured using the BioSpec-nano. cDNA was synthesized using PrimeScript RT Master Mix, and qRT-PCR was performed with SYBR Green Master Mix on a CFX Connect system. β-actin served as the internal reference, and the gene expression was calculated using the 2^−ΔΔCt^ method. The primers, synthesized by Shanghai Sangon Biological Engineering Co., Ltd. (Shanghai, China), are listed in Table 1.

### 2.5. The Microbiota Analysis

The fecal samples from the colons were sent to Baiqu Medical Technology Co., Ltd. (Shanghai, China) for 16S rRNA high-throughput sequencing. The process involved extracting the total DNA from 0.5 g of stool using a DNA extraction kit, followed by amplifying the V3-V4 region of the 16S rRNA gene using the primers 338F (5′-ACTCCTACGGGAGGCAGCAG-3′) and 806R (5′-GGACTACHVGGGTWTC TAAT-3′). Sequencing was conducted on an Illumina MiSeq platform using QIIME 2. The data underwent denoising via DADA2 and clustering at 100% sequence identity to generate the final Amplicon Sequence Variant (ASV) list. After normalization of the data, the samples were used for further analyses, including the alpha and beta diversity, the linear discriminant analysis effect size (LEfSe), and functional predictions. Taxa exhibiting statistically significant differences across groups were identified using the Kruskal–Wallis rank sum test (*p* < 0.05), with adjustment of the false discovery rate (FDR) via the Benjamini–Hochberg method (*q <* 0.05) to control for multiple comparisons.

### 2.6. Non-Targeted Metabolomics Analysis

The colon fecal samples were analyzed using LC-MS/MS at Baiqu Biomedical Technology (Shanghai). The tissue samples (25 ± 1 mg) were mixed with beads and 500 μL of extraction solution (MeOH: ACN:H_2_O = 2:2:1, with deuterated internal standards) and vortexed for 30 s. The analysis used a UHPLC system (Vanquish, Thermo Fisher) with a Waters BEH Amide column coupled to an Orbitrap Exploris 120 MS. The mobile phases were (A) 25 mmol/L ammonium acetate/ammonia hydroxide (pH = 9.75) and (B) acetonitrile. Injections of 2 μL were used at 4 °C. The MS was operated in IDA mode using Xcalibur software (Thermo Fisher Scientific, Waltham, MA, USA). under the following ESI conditions: sheath gas: 50 Arb; aux gas: 15 Arb; capillary temp: 320 °C; resolution: 60,000 (MS) and 15,000 (MS/MS); collision energy: 20/30/40; spray voltage: ±3.8/3.4 kV. The data were processed on a cloud platform and matched against the HMDB and KEGG databases, and metabolites were identified using the MS2 scores for reliability.

### 2.7. The Statistical Analysis

The data analysis and visualization were performed using SPSS 25.0 (SPSS Inc., Chicago, IL, USA) and GraphPad Prism 9.4.0 (GraphPad Software, San Diego, CA, USA). The results are presented as the mean ± the standard error of the mean (SEM). A one-way ANOVA and Student’s *t*-test were used to determine statistical differences between groups. Statistical significance was marked as “*” (*p* < 0.05), “**” (*p* < 0.01), and “***” (*p* < 0.001) compared to the CK group. Identical letters (a-c) within a column indicate significant mean differences (*p* < 0.05).

## 3. Results

### 3.1. The Phylogenetic Analysis and Genomic Features of EC-520

**Figure 1:**
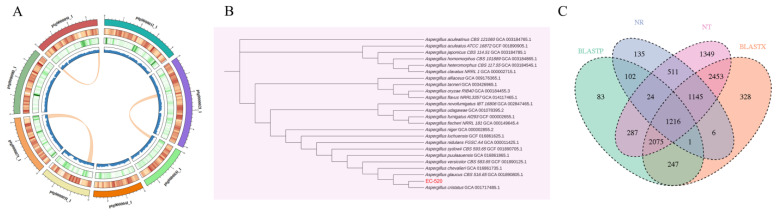
Genome-wide information, genetic evolution analysis, and functional annotation of EC-520. (**A**) A circular genome map (From the outer to inner circle, information is displayed as follows: chromosomes, gene density, repeat density, and GC content. The internal section displays collinear gene blocks within the genome identified through the MCScanX analysis. Each linking line in the center of the circle connects pairs of homologous genes.); (**B**) the neighbor-joining phylogenetic tree; and (**C**) the multi-database-annotated Venn diagram.

Whole genome sequencing and a comprehensive bioinformatic analysis were employed for the investigation of the genomic features of EC-520. The results showed that the total assembly size of the genome was 28,370,337 bp, the GC content was 46.67, Contig N50 was 3,578,266 bp, the number of assembled contigs was 9, and the BUSCO completeness was 97.3%, indicating high-quality assembly. Annotation identified 8453 protein-coding sequences (CDSs), with average gene and CDS lengths of 1907.38 bp and 1561.81 bp, respectively. Repetitive sequences accounted for 11.25% of the genome (3,192,757 bp). In addition, 227 tRNA, 38 rRNA, and 14 snRNA coding sequences were also identified (Figure 1A and Table 2). The complete genome sequence was submitted to the National Center for Biotechnology Information (NCBI) database under accession number GCA_044706195.1.

A neighbor-joining phylogenetic tree based on the mash distance matrix was built to reveal the exact phylogenetic position of EC-520 within 23 Aspergillus species. The closest evolutionary relative of EC-520 was *Aspergillus cristatus* GCA_001717485.1, but *Aspergillus niger* GCA_000002855.2 and *Aspergillus flavus* NRRL3357 GCA_014117465.1 were relatively distant strains (Figure 1B).

### 3.2. Analysis of the EC-520 Genome Annotation Results

The gene sequences of EC-520 were functionally annotated using the NT, NR, UniProt-BLASTX, and UniProt-BLASTP databases. Among the annotated genes, 3140 matched entries in NR, 9060 in NT, 7471 in UniProt-BLASTX, and 4035 in UniProt-BLASTP. The Venn diagram revealed 1216 genes shared across all four databases, while unique annotations included 135 (NR), 1349 (NT), 328 (UniProt-BLASTX), and 83 (UniProt-BLASTP) genes (Figure 1C).

### 3.3. The Effects of Different Doses of EC-520 on Growth in Rats

As depicted in Figure S1, the EC-520 treatment did not significantly affect the rats’ gain in body weight or organ morphology compared to those in the CK group. Furthermore, no notable differences were observed in the organ indices of the heart, liver, spleen, brain, lungs, or kidneys (Table 3).

### 3.4. The Effects of EC-520 on the Rats’ Colon Histomorphology and Barriers

**Figure 2:**
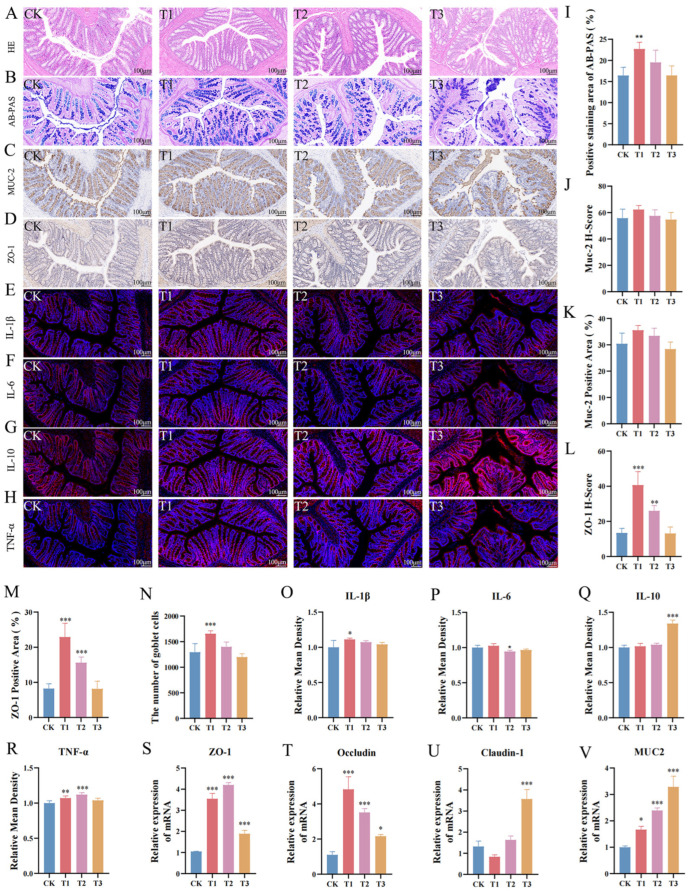
Effects of EC-520 on rat colon morphology and barrier function. (**A**,**B**) H&E and AB-PAS staining; (**C**,**D**) IHC analysis of Muc2 and ZO-1; (**E**–**H**) immunofluorescence staining images for IL-1β, IL-6, IL-10, and TNF-α; (**I**) Alcian blue–periodic acid–Schiff stain (AB-PAS)-positive areas; (**J**) Muc2 histochemistry scores (H-score); (**K**) Muc2-positive areas; (**L**) ZO-1 H-scores; (**M**) ZO-1-positive areas; (**N**) goblet cell counts; (**O**–**R**) IL-1β, IL-6, IL-10, and TNF-α levels; and (**S**–**V**) mRNA levels of ZO-1, Occludin, Claudin-1, and Muc2. Data: mean ± SEM (n = 5). Statistical significance was denoted as * (*p* < 0.05), ** (*p* < 0.01), and *** (*p* < 0.001) vs. CK group.

The histological analysis revealed an intact colon tissue structure across all groups, with a well-organized villus epithelium and a preserved gland architecture (Figure 2A). The AB-PAS staining demonstrated that EC-520 increased the goblet cell numbers and mucin levels in the colon, with a significant difference in the T1 group (*p* < 0.05) (Figure 2B). Figure 2C,D show that EC-520 up-regulated the expression of ZO-1 and Muc2 in the colon. Figure 2J–M indicate that compared to those in the CK group, both the histochemistry score (H-score) and the area of positive staining for the ZO-1 and Muc2 proteins were elevated in the T1 and T2 groups, with a significant increase in ZO-1 levels (*p* < 0.05). No significant differences were observed between the T3 group and the CK group. In addition, qRT-PCR confirmed the enhanced mRNA expression of ZO-1, Occludin, Claudin-1, and Muc2 in the T1, T2, and T3 groups (Figure 2S–V).

The immunofluorescence analysis of colonic cytokines revealed distinct expression patterns among the treatment groups. The T1 group showed significantly elevated IL-1β expression compared to that in the other groups (*p* < 0.05) (Figure 2E,O), while the IL-6 levels were markedly reduced in T2 (*p* < 0.05) (Figure 2F,P). The IL-10 expression was highest in the T3 group (*p* < 0.05), with no significant differences noted among the other groups (Figure 2G,Q), and the TNF-α levels were significantly higher in T1 and T2 versus those in CK (*p* < 0.05), with no change observed in T3 (Figure 2H,R).

### 3.5. The Effects of EC-520 on the Colon Microbiota in Rats

**Figure 3:**
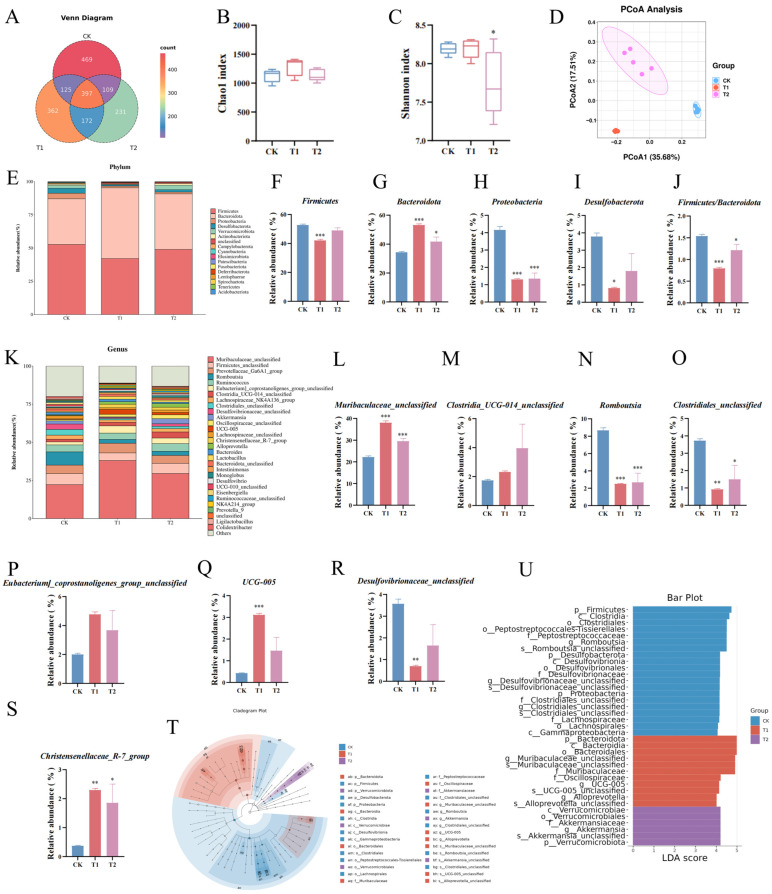
The 16S rRNA sequencing results in the feces from the colons of the EC-520-treated rats. (**A**) An ASV-level Venn diagram; (**B**) the Chao 1 index; (**C**) the Shannon index; (**D**) the PCoA; (**E**) phylum-level microbiota profiling; (**F**–**I**) the relative abundance of four phyla; (**J**) the Firmicutes/Bacteroidota ratio; (**K**) top 30 genera; (**L**–**S**) the relative abundance of eight genera; (**T**) the LEfSe cladogram; (**U**) the LEfSe bar (LDA > 4). Data: means ± SEMs (n = 5). Statistical significance was denoted as * (*p* < 0.05), ** (*p* < 0.01), and *** (*p* < 0.001) vs. CK group.

Based on the results of colon histomorphology, colon fecal samples from the T1, T2, and CK groups, which exhibited significant differences (no dose correlation was shown in T3), were selected for 16S rRNA gene sequencing to analyze the effects of the EC-520 treatment on the rat colon microbiota. The Venn diagram showed 397 shared ASVs, with 469, 362, and 231 unique ASVs in the CK, T1, and T2 groups, respectively (Figure 3A). The Chao 1 and Shannon rarefaction curves indicated a sufficient sequencing depth to capture most bacterial communities (Figures S2 and S3). The α-diversity analysis revealed an increasing trend in the Chao 1 index for the T1 and T2 groups (Figure 3B), though this was not significant, while the Shannon index significantly decreased in T2 compared to that in CK (Figure 3C), suggesting that the EC-520 intervention had no substantial impact on the richness of the microbes in the rats’ colons, while indicating a slight decrease in bacterial diversity. Good’s coverage exceeded 99.9% across the groups, confirming an adequate sequencing depth (Figure S4). The PCoA based on the Bray–Curtis distance showed significant differences in the colon microbiota structure among the groups (Figure 3D).

At the phylum level (Figure 3E), *Firmicutes*, *Bacteroidota*, *Proteobacteria*, and *Desulfobacterota* were the four most abundant phyla. Notably, the T1 group showed a significantly reduced abundance of *Firmicutes* versus that in the other groups (*p* < 0.05). Both the T1 and T2 groups exhibited decreased levels of *Proteobacteria*, *Desulfobacterota*, *Campylobacterota*, *Cyanobacteria*, and *Fusobacteriota* compared to those in CK, while the abundance of *Bacteroidota* was significantly elevated (*p* < 0.05). Consequently, the F/B ratio in the T1 and T2 groups was markedly lower than that in CK (Figure 3F–J).

Additionally, at the genus level (Figure 3K), *Muribaculaceae_unclassified*, *Firmicutes_unclassified*, *Prevotellaceae_Ga6A1_group*, *Romboutsia*, and *Ruminococcus* dominated the microbiota. The T1 and T2 groups showed a significantly increased presence of *Muribaculaceae_unclassified* and *Christensenellaceae_R-7_group* (*p* < 0.05) but a decreased presence of *Clostridiales_unclassified* and *Romboutsia* versus those in CK (*p* < 0.05). *Desulfovibrionaceae_unclassified* was reduced while *Clostridia_UCG-014_unclassified*, *Eubacterium_coprostanoligenes_group_unclassified*, and *UCG-005* were elevated in the T1/T2 groups (Figure 3L–S).

As shown in Figure 3T,U, the LEfSe analysis (LDA > 4) also proved that *p__Bacteroidota*, *f__Muribaculaceae* (*g__Muribaculaceae_unclassified*), *g__Alloprevotella*, and *f__Oscillospiraceae* (*g__UCG-005*) were the main microbiota in the T1 group. In contrast, the dominant bacterial taxa in the T2 group included *p__Verrucomicrobiota* and *f__Akkermansiaceae* (*g__Akkermansia*). Conversely, *p__Firmicutes*, *f__Peptostreptococcaceae* (*g__Romboutsia*), *c__Clostridia* (*o__Clostridiales*, *g__Clostridiales_unclassified*), *p__Desulfobacterota* (*g__Desulfovibrionaceae_unclassified*), *p__Proteobacteria*, *o__Lachnospirales* (*f__Lachnospiraceae*), and *c__Gammaproteobacteria* were enriched in the CK group.

### 3.6. The Effects of EC-520 on Colonic Fecal Metabolites in Rats

**Figure 4:**
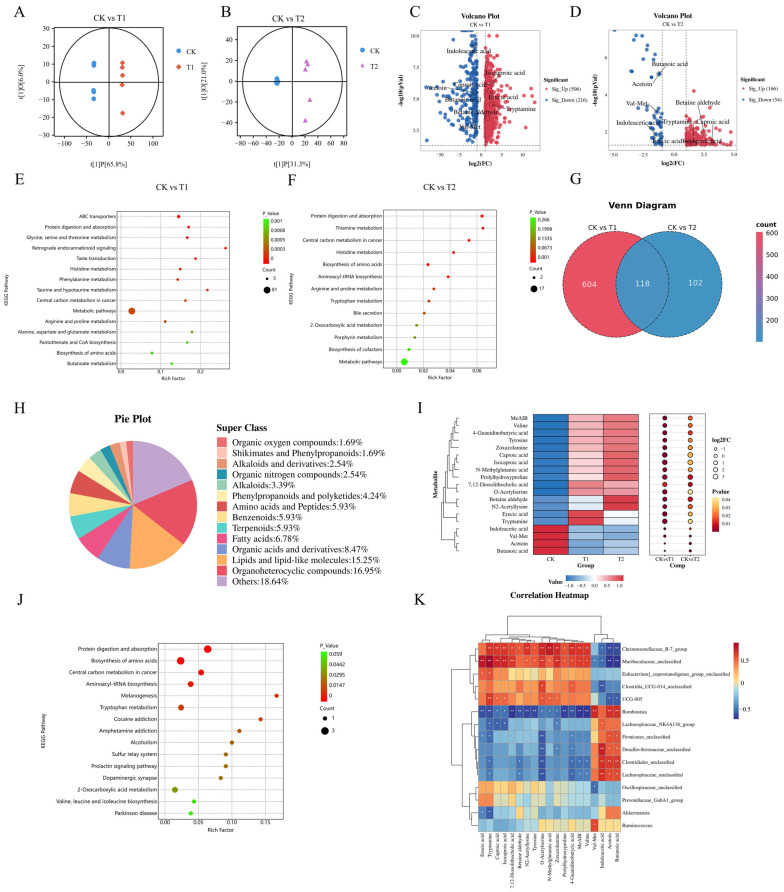
Effects of EC-520 on rat colon fecal metabolism and Spearman’s correlation analysis. (**A**,**B**) OPLS-DA of CK vs. T1 and CK vs. T2; (**C**,**D**) volcano plots of differential metabolites (FC > 2 or <0.5, VIP > 1, and *p* < 0.05); (**E**,**F**) KEGG enrichment pathways; (**G**) Venn diagram of shared metabolites; (**H**) pie chart of shared metabolites; (**I**) heatmap of 19 key metabolites; (**J**) KEGG pathways of key metabolites; and (**K**) Spearman’s correlation of core microbiota and metabolites. Data: means ± SEMs (n = 5). *, *p* < 0.05; **, *p* < 0.01.

The non-targeted metabolomics analysis of feces from the rat colons identified a total of 2200 metabolites. The predominant super classes of metabolites included organoheterocyclic compounds (22.53%), lipids and lipid-like molecules (17.93%), organic acids and derivatives (11.66%), benzenoids (10.94%), and alkaloids (4.96%) (Figure S5). The orthogonal partial least squares discriminant analysis (OPLS-DA) showed high discrimination and predictive capabilities, with R2Y = 1 and Q2 = 0.978 for the CK vs. T1 groups and R2Y = 0.997 and Q2 = 0.800 for the CK vs. T2 groups (Figures S6 and S7). The OPLS-DA score plot revealed that the CK group was dispersed across two distinct regions along the X-axis compared to the T1 and T2 groups, indicating increased inter-group differences and reduced intra-group variability (Figure 4A,B).

Fold change and VIP values were utilized to identify differential metabolites. Metabolites were classified as differential when they met the criteria of an FC > 2 or <0.5, a VIP > 1, and *p* < 0.05. The volcanic map results indicated that there were 722 distinct metabolites between the CK and T1 groups, with 506 metabolites up-regulated and 216 down-regulated (Figure 4C). Additionally, 220 different metabolites were identified between the CK and T2 groups, comprising 166 up-regulated and 54 down-regulated metabolites (Figure 4D). The KEGG enrichment analysis showed that pathways such as protein digestion and absorption, histidine metabolism, central carbon metabolism in cancer, and amino acid biosynthesis overlapped between the two comparison groups (Figure 4E,F).

The Venn diagram revealed a total of 118 distinct metabolites across the two comparison groups (Figure 4G), including 20 organic heterocyclic compounds, 18 lipids and lipid molecules, 10 organic acids and derivatives, 8 fatty acids, 7 amino acids and peptides, 7 benzenoids, 7 terpenoids, 5 phenylpropanoids and polyketides, 4 alkaloids, 3 alkaloids and derivatives, 3 organic nitrogen compounds, 2 organic oxygen compounds, and 2 shikimates and phenylpropanoids, along with 22 other metabolites (Figure 4H). Nineteen predominant metabolites with the highest MS2 scores were selected for further analysis, of which fifteen metabolites were found to be up-regulated in the T1 and T2 groups. These included tryptamine, caproic acid, isocaproic acid, erucic acid, tyrosine, valine, prolylhydroxyproline, O-acetylserine, N-methylglutamic acid, N2-acetyllysine, MeAIB, and betaine aldehyde. These substances were primarily amino acids and fatty acids. Conversely, four metabolites were down-regulated: indoleacetic acid, butanoic acid, Val-Met, and acetoin (Figure 4I). The different metabolites were significantly enriched in protein digestion and absorption, biosynthesis of amino acids, the central carbon metabolism in cancer, and tryptophan metabolism pathways (Figure 4J).

### 3.7. The Microbiota–Metabolite Correlation Analysis

The Spearman’s analysis revealed significant associations between the core gut microbiota and key metabolites (Figure 4K). Muribaculaceae_unclassified, Christensenellaceae_R-7_group, Eubacterium_coprostanoligenes_group_unclassified, Clostridia_UCG-014_unclassified, and UCG-005 were positively correlated with erucic acid, tryptamine, caproic acid, isocaproic acid, 7,12-dioxolithocholic acid, betaine aldehyde, N2-acetyllysine, and tyrosine and negatively correlated with butanoic acid, indoleacetic acid, val-met, and acetoin. Meanwhile, Romboutsia, Lachnospiraceae_NK4A136_group, Firmicutes_unclassified, Desulfovibrionaceae_unclassified, Clostridiales_unclassified, and Lachnospiraceae_unclassified had the opposite correlations with the above metabolites. Notably, the correlation between Muribaculaceae_unclassified and tryptamine was the most significant.

## 4. Discussion

In this study, the EC-520 strain derived from FBT was analyzed and identified using whole genome sequencing technology. It was found to be closely related to *Aspergillus cristatus* GCA_001717485-1 while showing a distant relationship with *Aspergillus flavus* NRRL3357 GCA_014117465.1, which is a harmful fungus. The results indicated that EC-520 could enhance the colon barrier function in rats, increase beneficial bacteria such as *Bacteroidota*, decrease potentially harmful bacteria such as *Proteobacteria* and *Desulfobacterota*, and significantly alter many metabolites, such as fatty acids and amino acids. The pathways of significant enrichment for these different metabolites were primarily associated with immune regulation, energy metabolism, and substance synthesis in rats. The Spearman’s correlation analysis showed that there was a significant correlation between the differential colon microbiota (*Muribaculaceae_unclassified* and *Clostridia_UCG-014_unclassified*) and the differential metabolites (erucic acid, tryptamine, and caproic acid). These results suggested that EC-520 might influence the secretion of metabolites by regulating the abundance of the colon microbiota, thereby playing a crucial role in enhancing colon barrier function.

As the predominant fungus in FBT, EC has been identified to possess several beneficial effects, including antioxidant properties, anti-tumor activity, and weight loss and lipid-lowering effects. However, its morphological similarity to *Aspergillus flavus* has led to some skepticism regarding its efficacy. With ongoing advancements in genome sequencing technology and bioinformatics analyses, numerous studies have reported on the whole genome sequencing and analysis of *Aspergillus* species [20]. Nevertheless, there remains a scarcity of research focusing on the whole genome sequence of EC. In the present study, we constructed the genome sequence of the EC-520 strain, which aligned closely with the genetic information for *Aspergillus cristatus* previously reported [18]. Our findings indicated that EC-520 was most closely related to *Aspergillus cristatus* (GCA_001717485-1) while also showing distant genetic affinity to *Aspergillus niger* GCA_000002855.2 and *Aspergillus flavus* NRRL3357 GCA_014117465.1. From an evolutionary perspective, our results suggested that the safety of EC-520 was linked to its beneficial effects. Furthermore, a total of 9060, 3140, 7471, and 4035 genes were annotated to the NT, NR, UniProt-BLASTX, and UniProt-BLASTP databases, respectively, providing a foundation for identifying the functional genes associated with EC-520. Compared with those in the CK group, there was no significant difference in the body weight or indexes of any of the important organs (the heart, liver, spleen, brain, lungs, and kidneys), indicating that EC-520 may have good safety under the current dosage and treatment methods, which was consistent with previous studies [21].

Tight junction proteins (ZO-1, Occludin, and Claudin-1) are crucial to intestinal integrity [22]. ZO-1 acts as a scaffold, Occludin regulates permeability, and Claudin-1 maintains barrier function and cell polarity [23,24,25]. Muc2, a key mucin, protects against microbial invasion [26]. qRT-PCR showed that EC-520 significantly increased the mRNA levels of ZO-1, Occludin, Claudin-1 and Muc2 in the colon tissue, aligning with the findings on EC polysaccharides’ protective effects [27]. EC-520 also boosted goblet cell numbers and mucin expression, enhancing mucosal barrier function [28]. In summary, EC-520 strengthens the tight junctions and Muc2 expression, improving the integrity of the mechanical colon barrier.

IL-1β, IL-6, and TNF-α are three pro-inflammatory cytokines that play crucial roles in the pathogenesis of inflammation [29], while IL-10 serves as an anti-inflammatory factor [30]. A normal inflammatory response is essential for activating both the local and systemic immune systems [31]. In this study, the expression of IL-10 in the T3 group was significantly increased, and the expression levels of TNF-α and IL-1β were increased in the T1 and T2 groups. The level of IL-6 in the T2 group was significantly reduced, suggesting that low-dose EC-520 stimulated and activated the anti-inflammatory mechanism in the body, thereby inhibiting a further inflammatory response and maintaining immune balance. Conversely, higher doses may be perceived by the body as a strong irritation or potential threat, triggering a pro-inflammatory response and increasing the expression of TNF-α and IL-1β, thereby mobilizing the immune system’s defense mechanisms. In addition, TNF-α up-regulates the expression of IL-1β by activating a specific signaling pathway while inhibiting the expression of IL-6, thus forming a complex cytokine regulatory network. This result is consistent with the dose-dependent role of the body’s immune response (high-dose immune activation, mid-dose balanced regulation, and low-dose anti-inflammatory dominance) [32,33].

The gut microbiota plays a vital role in immune system development and function [34]. When this balance is disrupted, it leads to alterations in the gut microbiome and deterioration of the immune system, rendering the host more susceptible to pathogens. In this study, the α-diversity analysis indicated an increasing trend in the richness of the colon microbiota through the EC-520 treatment. However, the diversity in the T2 group was significantly reduced, which was inconsistent with previous studies [35]. It is possible that the reduction in diversity was caused by partial degradation during the sample processing, and the detailed reasons for this degradation will be corrected in subsequent experiments. Furthermore, the β-diversity results demonstrated that EC-520 could modulate the composition and structure of the colon flora.

At the phylum level, EC-520 significantly increased the abundance of *Bacteroidota*, while it decreased the F/B ratio, which is an important indicator of the microbial balance in the colon and immune system enhancements [36]. This finding aligns with the fact that the exopolysaccharides of EC could reduce the F/B ratio and elevate the relative abundance of *Bacteroidota* [37]. Additionally, EC-520 reduced the abundance of *Proteobacteria*, *Desulfobacterota*, *Campylobacterota*, *Cyanobacteria*, and *Fusobacteriota*. These are potentially harmful bacteria [38,39,40,41,42]. At the genus level, the relative abundance of *Muribaculaceae_unclassified* and *Christensenellaceae_R-7_group* in the T1 and T2 groups was significantly increased compared to that in the CK group. The amplification of *Muribaculaceae_unclassified* is associated with intestinal immunity and is considered a beneficial component of the microbiome [43]. The *Christensenellaceae_R-7_group* primarily comprises Gram-negative bacteria and is thought to be closely related to reductions in inflammation [44]. Previous studies have shown that the EC fermentation can upregulate the abundance of *Christensenellaceae_R-7_group* [28], while the crude exopolysaccharides of *Aspergillus cristatus* and its purified substances can enhance the abundance of *Muribaculaceae* in mice [37]. These findings are consistent with the results of the present study. Conversely, the abundance of *Clostridiales_unclassified* and *Romboutsia* was significantly decreased in the T1 and T2 groups. An increased abundance of *Romboutsia* has been linked to higher cardiovascular risk and obesity [45], and certain members of *Clostridiales_unclassified* may exacerbate disease progression [46]. The relative abundance of *Desulfovibrionaceae_unclassified* in the T1 and T2 groups was lower than that in the CK group, while the relative abundance of *Eubacterium_coprostanoligenes_group_unclassified* was higher than that in the CK group. *Eubacterium_coprostanoligenes_group_unclassified* consists of bacteria capable of degrading cholesterol by converting it into coprosterol [47]. *Desulfovibrionaceae_unclassified* is an opportunistic pathogen associated with gastrointestinal diseases and colorectal cancer [48]. In summary, the EC-520 treatment resulted in an increase in the relative abundance of beneficial bacteria while decreasing the relative abundance of potentially harmful bacteria.

In addition to influencing the abundance of the colonic microbiota to maintain its balance, EC-520 can also induce changes in colonic metabolites, thereby facilitating their corresponding biological functions. In this study, compared to the CK group, 118 distinct metabolites were identified that were shared between the T1 and T2 groups, from which 19 predominant metabolites were selected, including 15 that were up-regulated, including tryptamine, caproic acid, isocaproic acid, erucic acid, tyrosine, valine, prolylhydroxyproline, O-acetylserine, N-methylglutamic acid, N2-acetyllysine, MeAIB, and betaine aldehyde. Conversely, four metabolites were found to be down-regulated: indoleacetic acid, butanoic acid, val-met, and acetoin. Notably, caproic acid is one of the products resulting from the fermentation of dietary fiber by intestinal microbes. The balance of the gut microbiome is essential for the proper functioning of the immune system, and caproic acid can promote the growth of beneficial bacteria [49]. Some studies have indicated that isocaproic acid and its derivatives, such as α-hydroxyisocaproic acid, play a role in anti-decomposition and promote muscle recovery in the human body, primarily by participating in amino acid metabolism [50]. Erucic acid has been shown to improve cognitive function, play a role in Huntington’s disease, interact with peroxisome proliferator-activated receptors, inhibit elastase and thrombin, and exhibit anti-inflammatory, antioxidant, and anti-tumor properties, as well as inhibit the influenza A virus [51]. Furthermore, EC-520 treatment can enhance the secretion of neurotransmitters and antioxidant-related amino acids, along with anti-inflammatory and antioxidant-related organic acids.

Tryptophan, an essential amino acid, has endogenous metabolites that are crucial for maintaining intestinal immune homeostasis and influence immune diseases. It is metabolized via three main pathways: the Kynurenine (Kyn) pathway, the 5-HT pathway, and the Indole pathway [52]. In the gastrointestinal tract, tryptamine stimulates the release of the neurotransmitter 5-HT from the intestinal chromaffin cells located on the mucosal surface, thereby enhancing gastrointestinal motility via the neurons of the enteric nervous system. Tissue perfusion studies of colon mucosa from mice have demonstrated that tryptamine can influence the ion secretion of the intestinal epithelial cells and is significant in regulating gastrointestinal motility [53]. Additionally, the metabolites produced by intestinal-microbiota-metabolizing amino acids, including tryptophan metabolites, are vital in host physiological and pathological processes. Notably, certain bacteria from the *Clostridium* genus have been shown to convert tryptophan into tryptamine [54]. In the present study, the Spearman’s correlation analysis showed that *Clostridia_UCG-014_unclassified* was positively correlated with tryptamine, indicating that *Clostridia_UCG-014_unclassified* might increase the production of tryptamine by affecting tryptophan metabolism and be interrelated with other metabolic pathways. This enables tryptophan metabolism to be closely integrated with the metabolic network of the entire organism, thereby realizing comprehensive regulation of its physiological functions. In addition, there was a significant positive correlation between *Muribaculaceae_unclassified* and tryptamine in this study. *Muribaculaceae* is a family of bacteria within *o_ Bacteroidota*. Members of this family produce short-chain fatty acids from both endogenous mucoglycans and exogenous polysaccharides, such as dietary fiber. *Muribaculaceae* exhibits a cross-feeding relationship with probiotics, including Bifidobacterium and Lactobacillus. Furthermore, it has been associated with reductions in inflammatory bowel disease, obesity, and type 2 diabetes [55]. Although direct evidence for *Muribaculaceae_unclassified* generating tryptamine was lacking, it was speculated that this family might be involved in the metabolism of amino acids in the gut. This involvement might influence metabolic processes such as amino acid deamination and decarboxylation, or it might regulate the utilization and transformation of amino acids through synergistic or competitive interactions with other microorganisms. For instance, it might impact the metabolism of branched-chain amino acids or aromatic amino acids, which play crucial roles in neurotransmitter synthesis and energy metabolism [56]. The Spearman’s correlation analysis revealed that significant positive correlations were observed between *Muribaculaceae_unclassified* and *Clostridia_UCG-014_unclassified* and erucic acid, caproic acid, and isocaproic acid. Consequently, this study suggested that EC-520 treatment might enhance the abundance of *Muribaculaceae_unclassified* and *Clostridia_UCG-014_unclassified* in the colon, subsequently increasing the secretion of tryptamine and influencing the release of 5-HT. Furthermore, EC-520 also increased the secretion of caproic acid, isocaproic acid, and erucic acid, playing a beneficial role in maintaining the balance of the colon microbiota and regulating immune function. The EC-520-induced microbiota–metabolite regulatory network may involve genomic function modules, but the specific targets and signaling pathways need to be verified through multi-omics integration studies.

## 5. Conclusions

In summary, the complete genome of EC-520 was 28,370,337 bp in length, and its genetic affinity indicated that it was distant from *Aspergillus flavus* NRRL3357 GCA_014117465.1. Notably, EC-520 enhanced the colon barrier and increased the abundance of *Muribaculaceae_unclassified* and *Clostridia_UCG-014_unclassified* in the colon, thus promoting the secretion of tryptamine and affecting the release of 5-HT. It also promoted the secretion of certain fatty acids, contributing to the maintenance of intestinal homeostasis. These findings provide robust support for the development of functional foods containing EC and new strategies for treating colon-related diseases.

## Figures and Tables

**Table 1:** Primer sequences.

Name	Forward Primer (5′->3′)	Reverse Primer (5′->3′)
ZO-1	AGCTGAGCTTTTCCTTCCGC	CGTCTCCTCAAAACCCTGCT
Muc2	GCCTCCTCTTGGCCTTGTAG	TCAGCACTCAGTGTTCGGTC
Occludin	CCCACTAGACCTTTCCATTGT	GACACAGCTTGTTCACTGCC
Claudin-1	TGGGGCTGATCGCAATCTTT	CTGGCATTGACAGGGGTCAT
β-actin	CTAAGGCCAACCGTGAAAAG	ACCAGAGGCATACAGGGACA

**Table 2:** EC-520 genome features.

Genome Features	Value
Genome size (bp)	28,370,337
GC content (%)	49.67
CDS (protein)	8453
Average gene length (bp)	1907.38
Average cds length (bp)	1561.81
Contig	9
Longest (bp)	5,088,186
N50 (bp)	3,578,266
N90 (bp)	2,640,403
BUSCO completeness (%)	97.3
Total length of the repeat sequence (bp)	3,192,757
5.8S rRNA	12
18S rRNA	26
tRNA	227
snRNA	14

**Table 3:** Effects of different EC-520 doses on the organ indexes of rats.

Group/Targets	Heart (%)	Liver (%)	Spleen (%)	Lung (%)	Kidney (%)	Brain (%)
CK	0.387 ± 0.021	3.744 ± 0.115	0.269 ± 0.015	0.660 ± 0.016	0.765 ± 0.017	0.672 ± 0.009
T1	0.373 ± 0.024	3.792 ± 0.125	0.290 ± 0.017	0.668 ± 0.042	0.794 ± 0.016	0.670 ± 0.017
T2	0.400 ± 0.020	3.795 ± 0.104	0.279 ± 0.018	0.697 ± 0.034	0.800 ± 0.014	0.672 ± 0.019
T3	0.361 ± 0.016	3.628 ± 0.109	0.264 ± 0.021	0.643 ± 0.030	0.784 ± 0.020	0.681 ± 0.011

The one-way ANOVA showed no significant differences among the groups (*p* > 0.05, n = 10).

## Data Availability

The original contributions presented in this study are included in the article; further inquiries can be directed to the corresponding author.

## Supplementary Materials

The following supporting information can be downloaded at https://www.mdpi.com/article/10.3390/foods14091569/s1. Figure S1: Body weight; Figure S2: Chao 1 dilution curve; Figure S3: Shannon dilution curve; Figure S4: Good’s coverage; Figure S5: Pie chart of metabolite classification; Figure S6: Comparison scoring chart between the CK group and the T1 group; Figure S7: Comparison scoring chart between the CK group and the T2 group.
