# Supplementary figures and images for: Genomic Characterization and Functional Evaluation of Eurotium cristatum EC-520: Impacts on Colon Barrier Integrity, Gut Microbiota, and Metabolite Profile in Rats
Source: Foods. 2025 Apr 29;14(9):1569. doi: 10.3390/foods14091569 (PMC12071741; doi:10.3390/foods14091569)

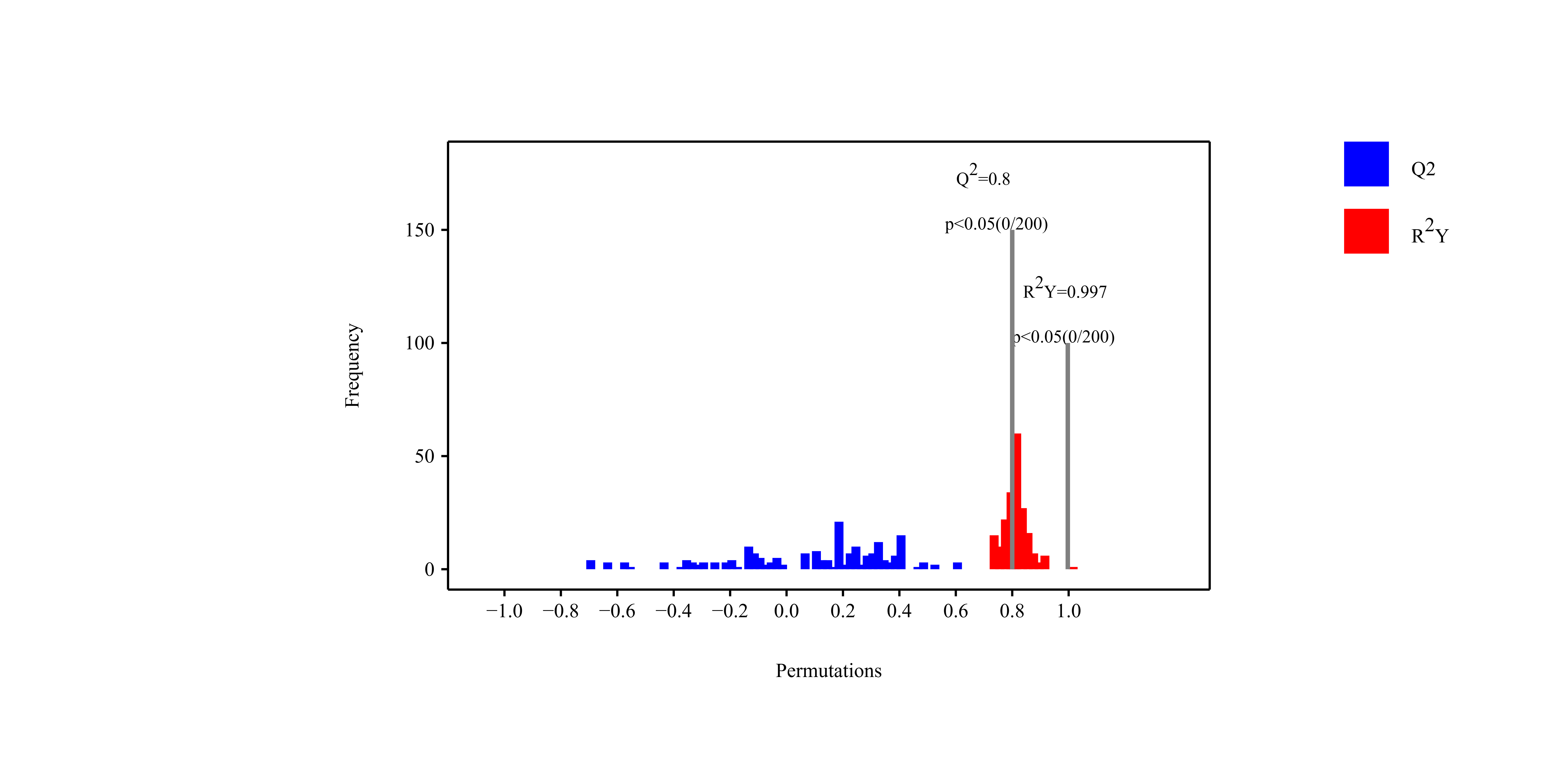

Supplement: Supplementary file 1 [file foods-14-01569-s001.zip › Figure S7.tif]

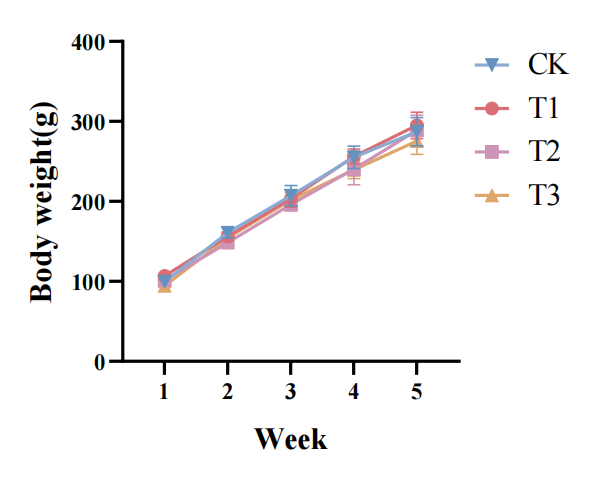

Supplement: Supplementary file 1 [file foods-14-01569-s001.zip › Figure S1.tif]

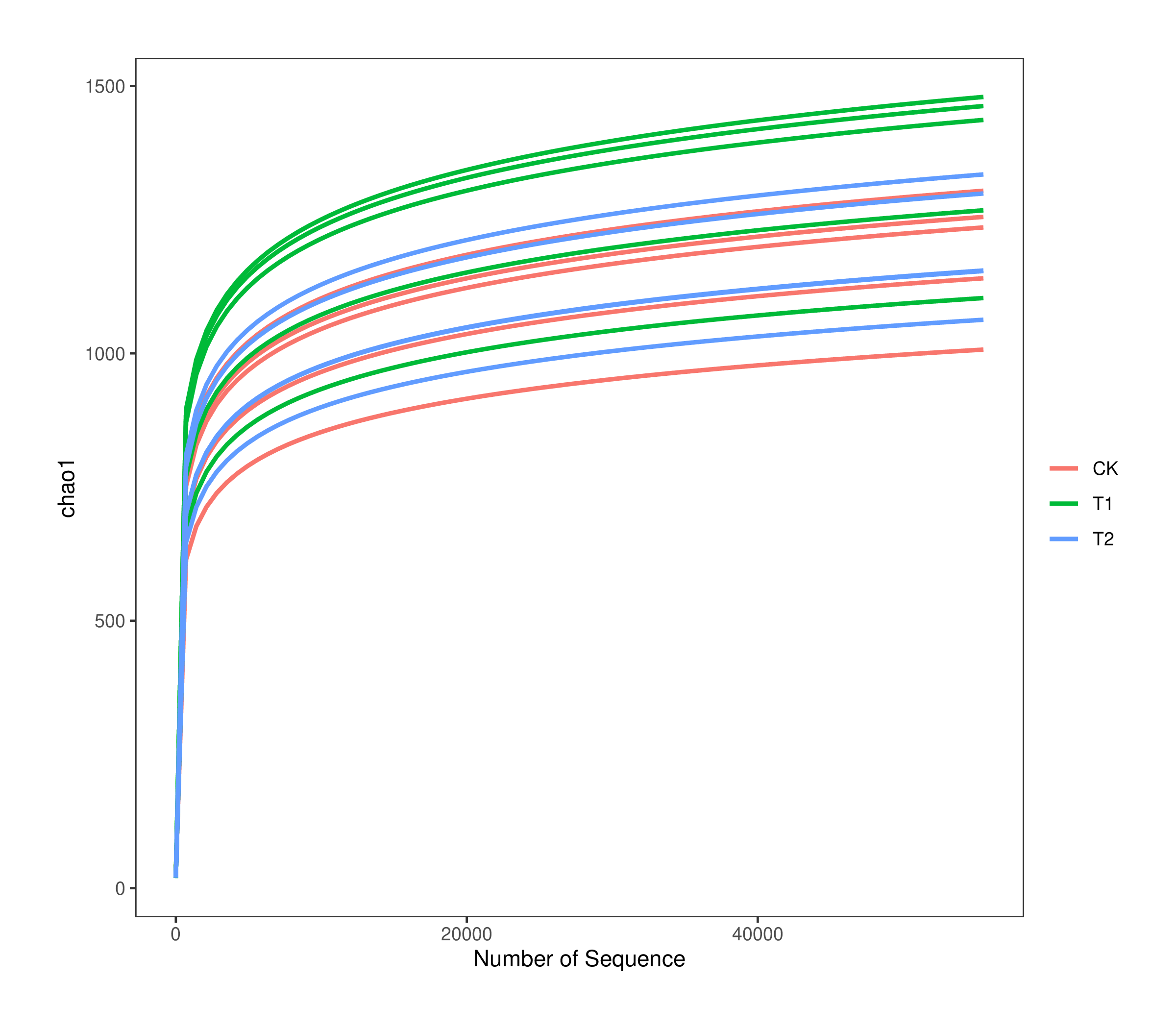

Supplement: Supplementary file 1 [file foods-14-01569-s001.zip › Figure S2.tif]

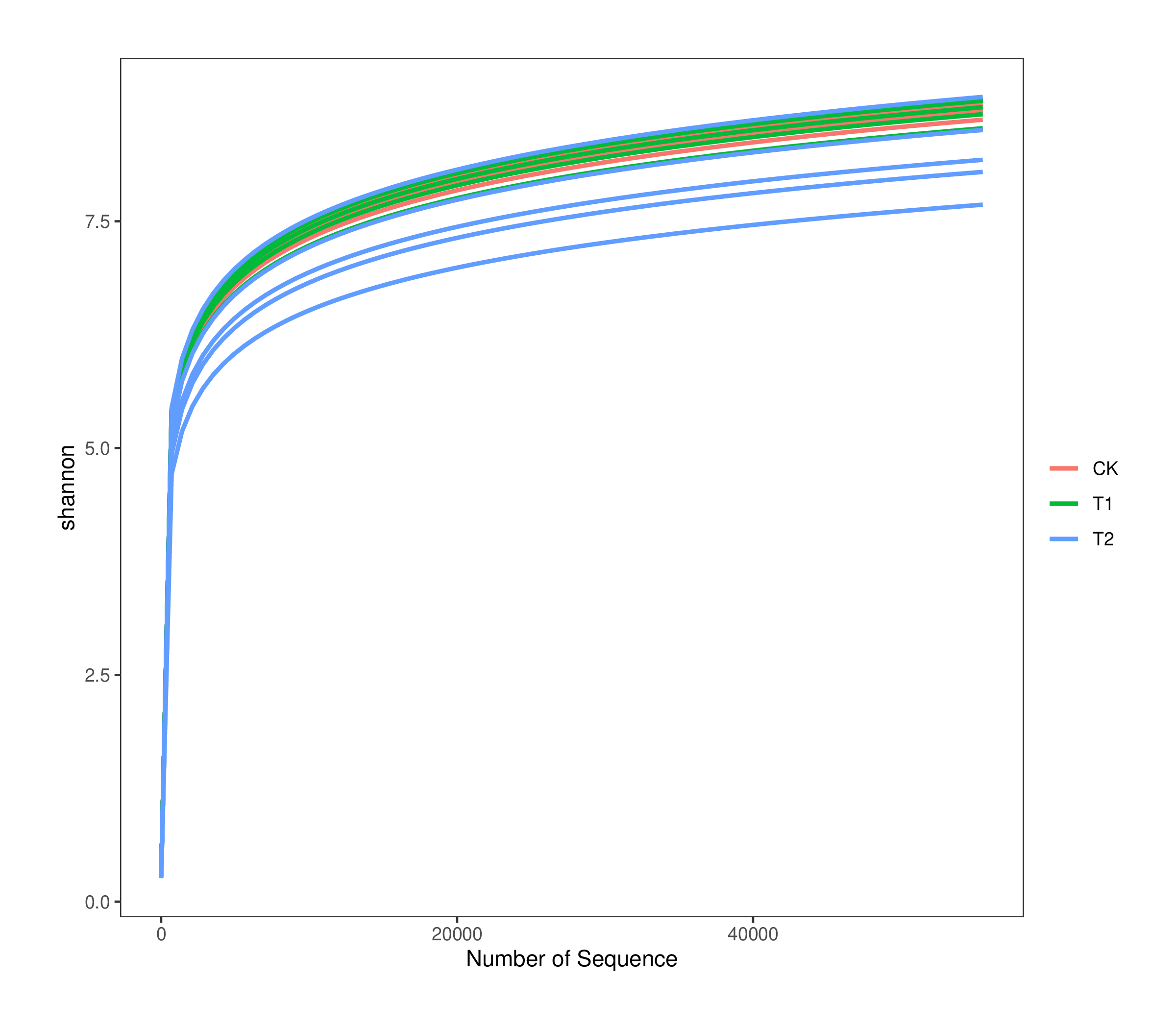

Supplement: Supplementary file 1 [file foods-14-01569-s001.zip › Figure S3.tif]

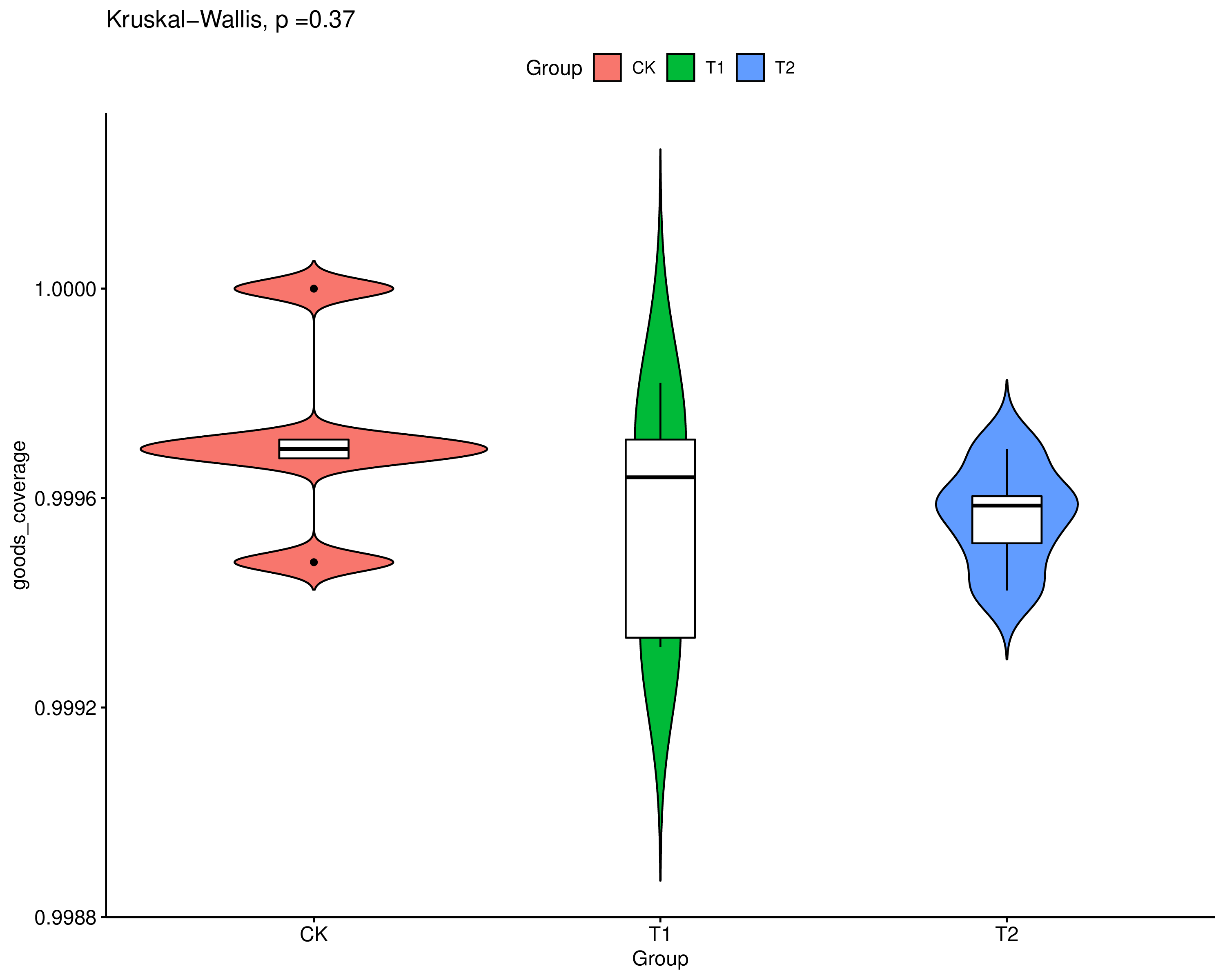

Supplement: Supplementary file 1 [file foods-14-01569-s001.zip › Figure S4.tif]

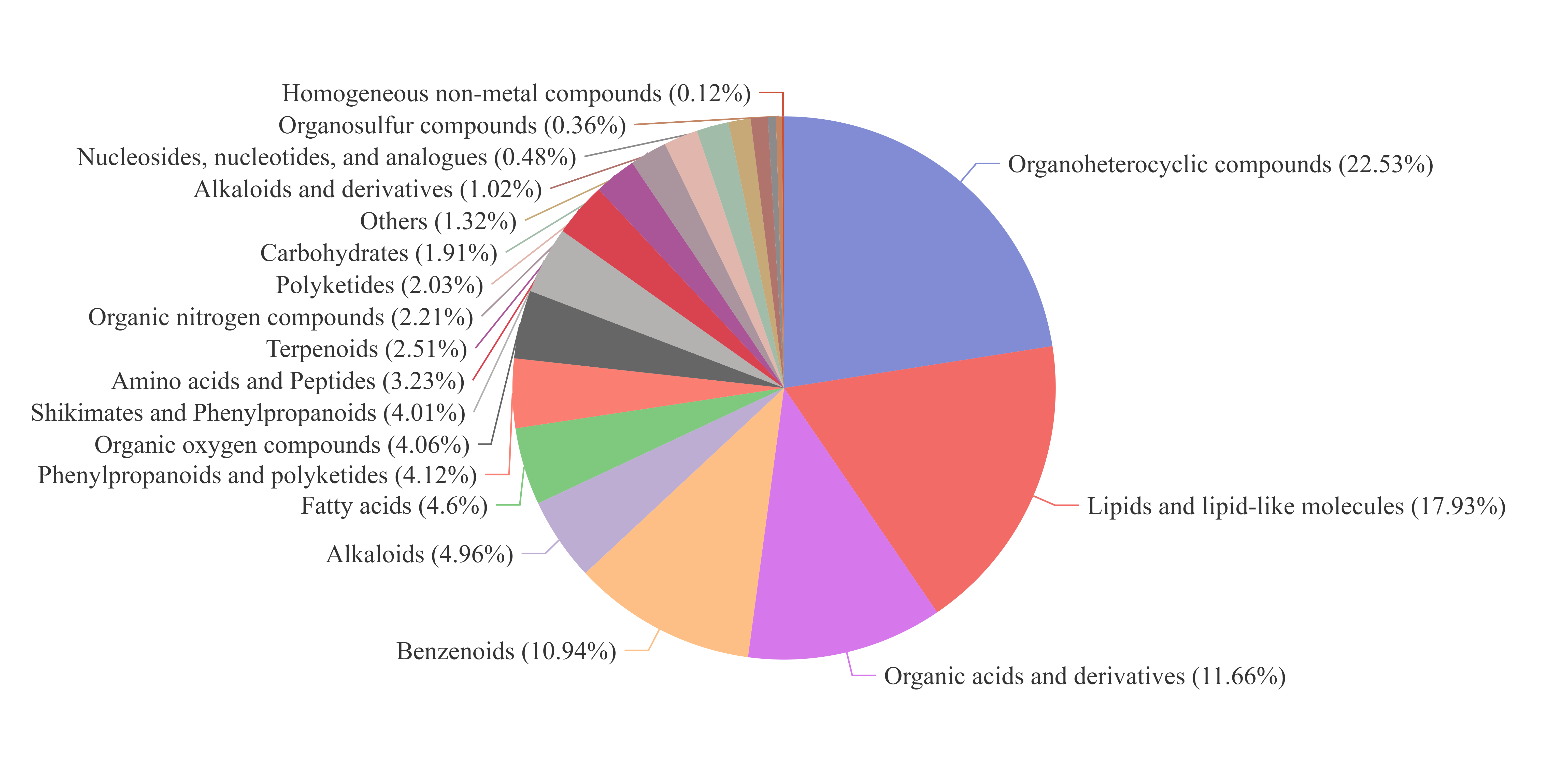

Supplement: Supplementary file 1 [file foods-14-01569-s001.zip › Figure S5.tif]

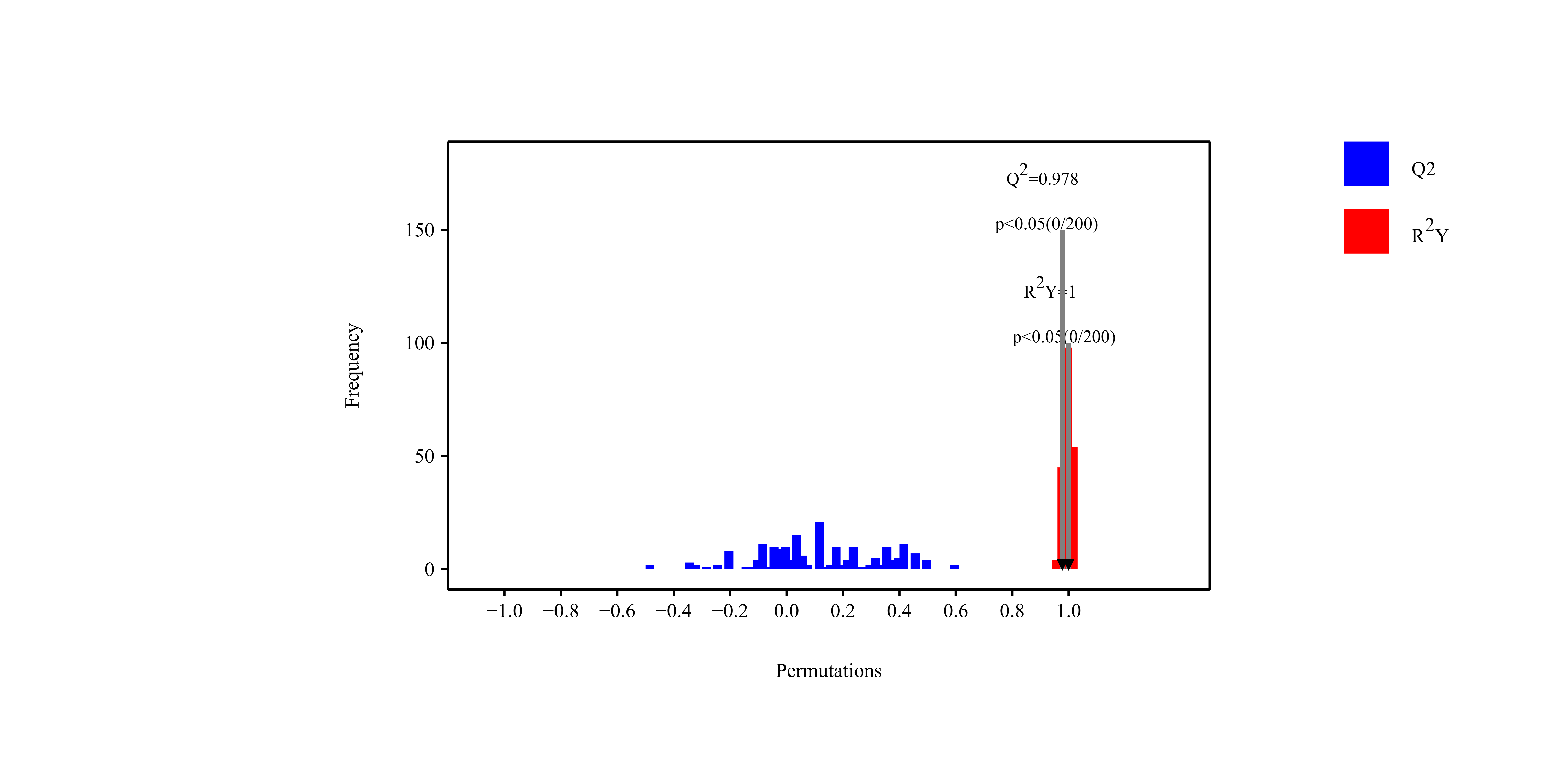

Supplement: Supplementary file 1 [file foods-14-01569-s001.zip › Figure S6.tif]
